# A fault-tolerant and robust controller using model predictive path integral control for free-flying space robots

**DOI:** 10.3389/frobt.2022.1027918

**Published:** 2022-12-07

**Authors:** Mehran Raisi, Amirhossein Noohian, Saber Fallah

**Affiliations:** ^1^ Connected and Autonomous Vehicles Laboratory, School of Mechanical Engineering Sciences, University of Surrey, Guildford, England; ^2^ Department of Mechanical Engineering, Sharif University of Technology, Tehran, Iran

**Keywords:** space robots, model predictive path integral control, space debris removal, parameter uncertainity, planner-estimator model predictive path integral controller

## Abstract

The use of manipulators in space missions has become popular, as their applications can be extended to various space missions such as on-orbit servicing, assembly, and debris removal. Due to space reachability limitations, such robots must accomplish their tasks in space autonomously and under severe operating conditions such as the occurrence of faults or uncertainties. For robots and manipulators used in space missions, this paper provides a unique, robust control technique based on Model Predictive Path Integral Control (MPPI). The proposed algorithm, named Planner-Estimator MPPI (PE-MPPI), comprises a planner and an estimator. The planner controls a system, while the estimator modifies the system parameters in the case of parameter uncertainties. The performance of the proposed controller is investigated under parameter uncertainties and system component failure in the pre-capture phase of the debris removal mission. Simulation results confirm the superior performance of PE-MPPI against vanilla MPPI.

## 1 Introduction

**FIGURE 1:**
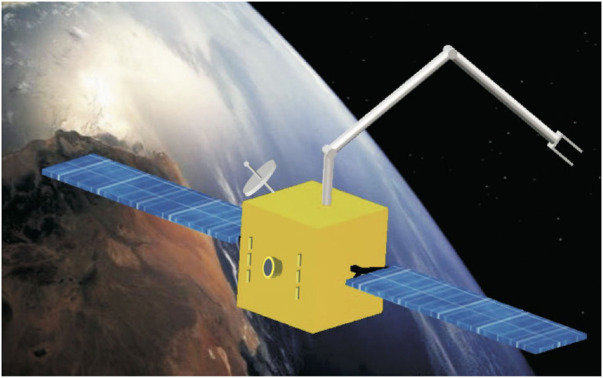
A schematic of a space manipulator system (Nanos and Papadopoulos, 2017).

The application of a space robot, a manipulator connected to a free-flying base, is becoming more popular as it can be extended to different space missions (Figure 1) (Nanos and Papadopoulos, 2017). Many space missions include several tasks such as inspecting, refueling, assembling and constructing, and removing space debris. Currently, these operations are performed by astronaut Extravehicular Activities (EVA). However, the risky nature of such operations can threaten astronauts’ life and require careful preparation. A suitable solution is performing such operations by space manipulators (Papadopoulos et al., 2021). Being small makes these manipulators perfect for moving around the main satellite with faster acceleration.

Small space robots such as the Future Space Debris Removal Orbital Manipulator (FSDROM) can play a significant role in future debris removal missions (Shyam et al., 2021). In 2019, the European Space Agency (ESA) claimed that over 8,000 tons of space debris are in Earth’s orbit, and this number is increasing. A large amount of space debris can cause risks to satellites and astronauts (Chatterjee, 2014). Therefore, removing space debris is becoming among the top concerns in space missions. One of the methods for removing space debris is through direct capture of objects. Direct capture methods are divided into rigid and flexible capture (Zhao et al., 2020). Several methods for flexible direct capturing, such as nets, harpoons, and tentacles, have been proposed (Billot et al., 2014; Zhang and Huang, 2016; Forshaw et al., 2017). Flexible capturing mechanisms reduce risks associated with the collision between space robots and target debris, which decreases the risk of unsuccessful captures that can generate more debris (Biesbroek et al., 2017). On the other hand, rigid capturing mechanisms are promising methods for controlling unknown objects, especially in detumbling phase, as they have more control over the object. In addition, they can be accomplished with available tools used in servicing missions, whereas flexible mechanisms need extra equipment.

Applying space robots in rigid debris removal missions is challenging since space debris are mainly non-cooperative moving objects that do not provide any information to track them. Several missions for on-orbit rigid capturing using space manipulators demonstrated their potential for future space missions. For instance, the Engineering Test Satellite VII “KIKU-7” (ETS-VII) by the Japan Aerospace Exploration Agency (JAXA) in 1997 was among the pioneers in demonstrating space robotic capability using chasers and target satellites (Yoshida, 2003). In 2016, Aolong-1 (ADRV) was developed by the China Academy of Launch Vehicle Technology (CALT) to investigate the removal of space debris in an experiment by grasping an object and sending it back on a re-entry trajectory. The satellite used a robotic arm to grasp space debris and throw them back into the atmosphere.

Space robots’ operation and performance in capturing space debris rely on their control systems. However, there are some concerns associated with the design of space robot control systems due to the following points:

Presently, the average lifespan of some satellites is approximately 14 years (Tomaszewska et al., 2019), and the maintenance of satellites is expensive. It means that the robot control law shall be adaptable to endure the change of system parameters during its mission.

Human intervention (telerobotics) in space missions is difficult. For instance, in Biesbroek et al. (2017) authors claimed the debris satellite ENVISAT is predicted to rotate around five degrees per second, and capturing such a massive object (about 8 tons) is beyond the human’s performance and needs expertise. In such cases, it is better to use high-performance controllers to handle space robots. Hence, autonomy in space missions is much preferred.

Space manipulators are in direct contact with unidentified rotating debris, and damage to the actuators and the robot’s structure is unavoidable. Therefore, any controlling law shall be sufficiently robust to maintain its performance in the possibilities of an actuator failure or malfunction. This event is more possible in the case of direct capturing methods where capturing of objects can cause large impacts on the spacecraft (Seweryn et al., 2022).

Accurate identification of system parameters is inevitable in rigid capturing missions where many parameters such as inertia, friction, geometry, and attitude must be identified to ensure the controller’s performance (Aghili, 2020).

Fulfilling such requirements through classical control approaches is not a trivial task due to their limitation in handling system uncertainties and contact modeling. Recently, model predictive control (MPC) for robot controls has received significant attention from academia and industry due to its benefits, such as the power to handle constraints (Hewing et al., 2020). This paper proposes a novel model-based controller based on Model Predictive Path Integral Control (MPPI) to approach debris sites under critical operating conditions. MPPI is a sampling-based algorithm that applies the iterative path integral control update law in a model predictive control setting (Williams et al., 2016). MPPI has demonstrated great performance in controlling highly nonlinear dynamic systems. However, its performance is sensitive to system parameters and dynamic uncertainties due to its reliance on the system’s dynamic model. To address this limitation, in this paper, we introduce a Planner-Estimator MPPI (PE-MPPI) framework to increase the controller’s robustness against model uncertainties and the change of parameters. This framework consists of two parts: Planner MPPI, which controls the free-flying space manipulator, and Estimator MPPI, which estimates on-board model parameters. In this regard, when the on-board model cannot track the real system, Estimator MPPI readjusts on-board model parameters to minimize the difference between the real and on-board models’ responses. Moreover, being sample-based helps MPPI change its on-board model with respect to actuator failures or malfunctioning alarms without any need to redesign the controller, which is a difficult task for classical control systems.

In the present study, we consider some assumptions to develop our method. Firstly, in the context of PE-MPPI all uncertainties are supposed to be structural, and unstructured uncertainties cannot be handled efficiently by the proposed algorithm. Secondly, we do not address directly the saturation problem of control effort. Instead, by defining a cost for actions we can indirectly penalize control efforts to be as small as possible.

This paper is structured as follows: Section II describes current state-of-the-art control systems and techniques for space robots in space missions. Section III explains the kinematics and dynamics formulation of space robots. In section IV, the MPPI algorithm is described. The extension to this algorithm, which is the main contribution of this paper, is then explained in section V. The simulation environment, robot operation scenarios, and simulation results are presented in section VI. Finally, the conclusions and future works are outlined in section VII.

## 2 Related works

Parameters of a space manipulator are reasonably measured and applied for controller design before launching to space. However, some parameters such as the joints’ damping coefficient and stiffness can change over time. Hence, on-orbit identification is required to guarantee the space robot’s performance (Zhao et al., 2020). Researchers in Yoshida (2003) developed a method for identifying the inertial parameters based on the conservation of momentum and the effect of gravity gradient torque. They validated their method by comparing results with data obtained from the ETS-VII Japanese space robot. Moreover, researchers in Christidi-Loumpasefski et al. (2017) proposed an agile adaptation law to identify all parameters of a free-floating space robot based on the conservation of angular momentum without any data from joint accelerations or torques.

Designing a motion-planning framework for space manipulators has been extensively investigated, taking into account dynamic coupling and singularities, as well as the physical restrictions of space robots. For instance, researchers attempted to solve the trajectory planning problem by minimizing a cost function that satisfies specific criteria, e.g., power consumption (Seweryn and Banaszkiewicz, 2008; Rybus et al., 2016). An effective motion planning strategy was proposed for a 6-DoF space robot based on Particle Swarm Optimization (PSO) to optimize the base berth position as an optimizable parameter (Zhang and Liu, 2018). Mu et al. proposed a unified modeling framework for multiple moving obstacles that was computationally efficient, as well as a collision-free trajectory planning approach for a redundant space manipulator (Mu et al., 2017).

Recently, reinforcement learning has received significant attention from robotic researchers due to its strength in controlling nonlinear dynamic systems. The reinforcement learning techniques can be classified as model-free and model-based techniques. The model-free techniques train a robot agent through interaction with the environment. Model-free reinforcement learning is a powerful technique in controlling complex dynamic systems as they do not use the model of the system. However, it suffers from sample efficiency and a long training time. Broida and Linares (2019) created a control strategy based on Proximal Policy Optimization (PPO) to bring one satellite into a docking position with another in a relative orbit reference frame. In Wu et al. (2020), proposed a trajectory planning methodology based on Deep Deterministic Policy Gradient (DDPG) for a dual-arm free-floating space robot. The proposed algorithm was able to approach both moving and fixed targets. There are some challenges regarding applying model-free reinforcement learning algorithms in the real world, such as slow learning rate and the cost of training in the real world, which makes transfer learning a suitable solution. In contrast, model-based reinforcement learning uses the model of the system to make the learning process faster and more efficient (Morgan et al., 2021).

Model predictive control (MPC) is an advanced control method that, similar to model-based reinforcement learning, uses a system model to predict the system’s future behavior. MPC solves an online optimization algorithm to find the optimal control action that drives the predicted output to the reference. One of the state-of-the-art model predictive control techniques is Model Predictive Path Integral Control (MPPI) (Williams et al., 2016). Being sampling-based and derivative-free makes MPPI an ideal candidate for convex and non-convex constraints, where gradient-based model predictive controllers suffer significantly (Williams et al., 2017b; Dixit et al., 2019). Moreover, MPPI’s performance depends considerably on the number of trajectories sampled using the on-board model, and the embedding computation can benefit from recent advances in Graphics Processing Units (GPUs) to achieve better real-time performance. It means one can adjust MPPI performance in real-world applications by selecting suitable processors (Arruda et al., 2017; Kim et al., 2022). MPPI has been used to control aerial and terrestrial robots (Williams et al., 2016; Pravitra et al., 2020). Various algorithms have been proposed to enhance MPPI performance. For example, the authors of (Lowrey et al., 2018) proposed combining MPPI with the concept of value function from model-free reinforcement learning to enhance the MPPI exploration phase. In some works, researchers worked on making MPPI robust to disturbances. In Williams et al. (2018), proposed Tube-MPPI by combining Tube-MPC and MPPI. The result was a robust algorithm that managed cost functions with discontinuous and sparse gradient information. In Gandhi et al. (2021), Gandhi et al. developed Robust MPPI (RMPPI) and investigated its performance on off-road navigation. The algorithm outperformed MPPI and Tube-MPPI in terms of agility and robustness to disturbances. Besides not being robust to disturbances, conventional MPPI’s performance is sensitive to the on-board model, an approximate model of the real system. Structural uncertainties like actuator specifications and lack of environment information like debris inertia can reduce MPPI’s performance. In Pravitra et al. (2020), authors combined MPPI control with L1-adaptive control, resulting in a multirotor controller which was robust to the changes in the system dynamics. L1-adaptive control robustified the architecture; therefore, the overall system behaved similarly to the nominal system with MPPI. In this paper, we propose Planner-Estimator MPPI (PE-MPPI), a novel planner-estimator framework based on the MPPI algorithm, which can handle the model’s uncertainties by minimizing the norm of the difference signal. When the on-board model cannot track the real system and the resultant error becomes greater than a threshold, PE-MPPI readjusts the on-board model parameters. The proposed framework will be further discussed in section V.

## 3 Prerequisites

### 3.1 Kinematics of a space robot

The kinematics of industrial manipulators depends only on the parameters of the joint space, whereas the kinematics of the space robots is more complex than terrestrial robots. The kinematics of a space robot is determined based on the position and orientation of the base and joint parameters.

**FIGURE 2:**
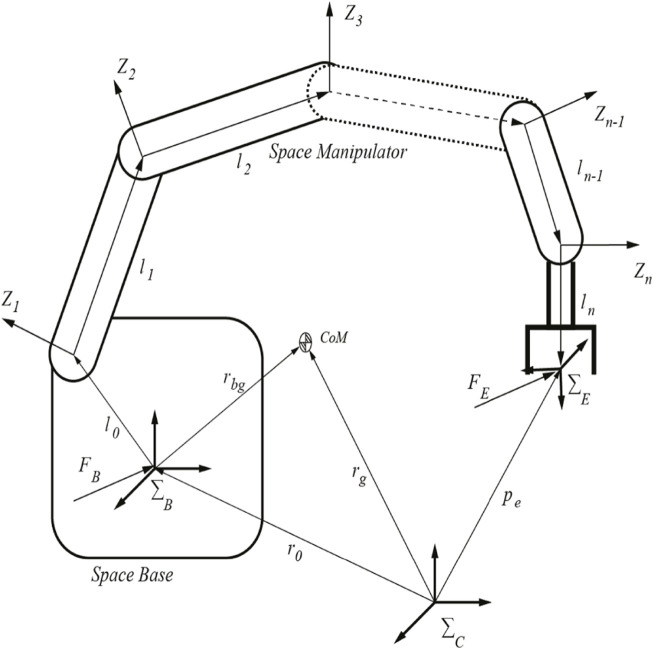
The configuration of a space robot and the coordinates of the joints.

According to Figure 2, the space robot can be represented as a set of n+1 rigid links connected with n joints, resulting in n+6 degrees of freedom. Furthermore, *Σ*_*C*_ is the inertial coordinates system, and *Σ*_*B*_ the base coordinates system attached on the base with its origin at the centroid of the base. Therefore, the position of the end-effector can be obtained as follows:

$$p_{e}=r_{0}+l_{0}+\overset{n}{\underset{i=1}{\sum }}l_{i}$$ (1)

where:

*p*_*e*_: The position vector of the end-effector in the coordinates system Σ_*C*_

*r*_0_: The position vector of the centroid of the base in the coordinates system Σ_*C*_

*l*_0_: The connection vector from the base to the first joint

*l*_*i*_: The connection vector from joint i to joint i+1.

By differentiating the kinematic equation with respect to time, the relation between the velocity of the end-effector and the velocity of the joints can be obtained as follows:

$${\dot{x}}_{e}=J_{m}\dot{\phi }+J_{b}{\dot{x}}_{b}$$ (2)

where:

${\dot{x}}_{e}$ : The linear/angular velocity of the end-effector in the inertial coordinates system.

$\dot{\phi }$ : The angular velocity of the joints.

${\dot{x}}_{b}$ : The linear/angular velocity of the base in the base coordinates system.

*J*_*m*_: The Jacobian matrix of the manipulator.

*J*_*b*_: The Jacobian matrix of the base.

### 3.2 Dynamics of a space robot

The dynamics of space robots are more complicated than terrestrial robots due to the dynamics coupling effect between the manipulator arm and its base. For instance, the space robot base would react based on the momentum conservation theorem if torque *τ*_*i*_ is applied to the *i*th joint (Huang et al., 2006). Accordingly, the center of mass of the whole structure relative to the *Σ*_*C*_ coordinates system would not change, but the *Σ*_*B*_ coordinates system would move. Determining the dynamics of the space robot is necessary to ensure the successful execution of missions. The equation of motion for a free-flying space robot with n links is as follows:

$$\left[\begin{array}{cc} H_{b} & H_{bm}\\ {H_{bm}}^{T} & H_{m} \end{array}\right]\left[\begin{array}{c} {\ddot{x}}_{b}\\ \ddot{\phi } \end{array}\right]+\left[\begin{array}{c} c_{b}\\ c_{m} \end{array}\right]=\left[\begin{array}{c} F_{b}\\ \tau \end{array}\right]+\left[\begin{array}{c} J_{b}^{T}\\ J_{m}^{T} \end{array}\right]F_{h}$$ (3)

where:

*H*_*b*_: The inertial matrix of the base.

*H*_*m*_: The inertial matrix of the manipulator arm.

*H*_*bm*_: The coupling inertial matrix between the base and the manipulator arm

*c*_*b*_: The velocity-dependent non-linear term of the base

*c*_*m*_: The velocity-dependent non-linear term of the manipulator arm.

*F*_*b*_: The force and torque on the centroid of the base.

*F*_*h*_: The force and torque on the end-effector

*τ*: The joint torque of the manipulator arm.

When no external forces are applied to the end-effector (*F*_*h*_ = 0), and the thrusters (or reaction wheels) do not apply force to the spacecraft base (*F*_*b*_ = 0), the above dynamic equation will be reduced to the following form:

$$H_{b}{\dot{x}}_{b}+H_{bm}\dot{\phi }=\left[\begin{array}{c} P\\ L \end{array}\right]=const.$$ (4)

where *p* and *L* are linear and angular momentums, which are constant values. The free-floating space robots are divided into two sub-types where the initial momentum is zero or no-zero (Nanos and Papadopoulos, 2011; Basmadji et al., 2020). In this study, the debris site is outside the reach of the spacecraft robot. Therefore, it is necessary to use the model of free-flying space robots in which thrusters and reaction wheels traverse in space.

## 4 Model predictive path integral control

Model predictive path integral control (MPPI) is an importance-sampling method. Its derivative-free behavior makes it an excellent choice for optimal control problems with nonlinear dynamics and non-convex cost functions. The fundamental notion of MPPI is to sample many trajectories for a time horizon of *T* from a dynamical system. Each trajectory *τ* = {*x*_0_, *u*_0_, *x*_1_, *u*_1_, … , *x*_*T*_, *u*_*T*_} is then evaluated according to a cost function. Accordingly, the optimal trajectory is computed based on its importance over all trajectories. To determine near-optimal solutions, increasing the number of trajectories is necessary. Fortunately, this can be quickly accomplished by taking advantage of the parallel nature of sampling and using Graphical Processor Unit (GPU) (Mohamed et al., 2020).

Consider a discrete-time dynamical system as follows:

$$x_{t+1}=f\left(x_{t},u_{t}+\delta u_{t}\right)$$ (5)

where *x*_*t*_ is the state vector, *u*_*t*_ is the control input vector, and *δu*_*t*_ is the random vector sampled from a zero-mean Gaussian distribution *N* (0, Σ_*u*_) at time-step t. As mentioned, each trajectory can be evaluated with a cost function as follows:

$$S\left(\tau \right)=\phi \left(x_{T}\right)+\overset{T}{\underset{t=0}{\sum }}q\left(x_{t},u_{t}\right)$$ (6)

where *ϕ*(*x*_*T*_) and *q* (*x*_*t*_, *u*_*t*_) are the terminal and running costs, respectively. MPPI aims to find the optimal control input trajectory u* = (*u*_0_, *u*_1_, … , *u*_*T*_), which minimizes the expectation over all generated trajectories as follows:

$$J=\min_{u}\left(E\left[S\left(\tau \right)\right]\right)$$ (7)

The solution to this problem has been discussed in Williams et al. (2017a). The authors used the Feynman-Kac lemma to turn this problem into a stochastic process. The consequent update law of the control input is as follows:

$$u_{t}\leftarrow u_{t}+\;\frac{\sum_{k=1}^{K}\exp\left(\left(\frac{-1}{\lambda }\right)\left[S_{k}\left(\tau \right)-\min\left(S\right)\right]\delta u_{t,k}\right)}{\sum_{k=1}^{K}\exp\left(\left(\frac{-1}{\lambda }\right)\left[S_{k}\left(\tau \right)-\min\left(S\right)\right]\right)}$$ (8)

where *K* is the number of trajectories, and *λ* is called inverse temperature. The detailed MPPI algorithm is described in Algorithm 1.

Algorithm 1 MPPI (Mohamed et al., 2020).

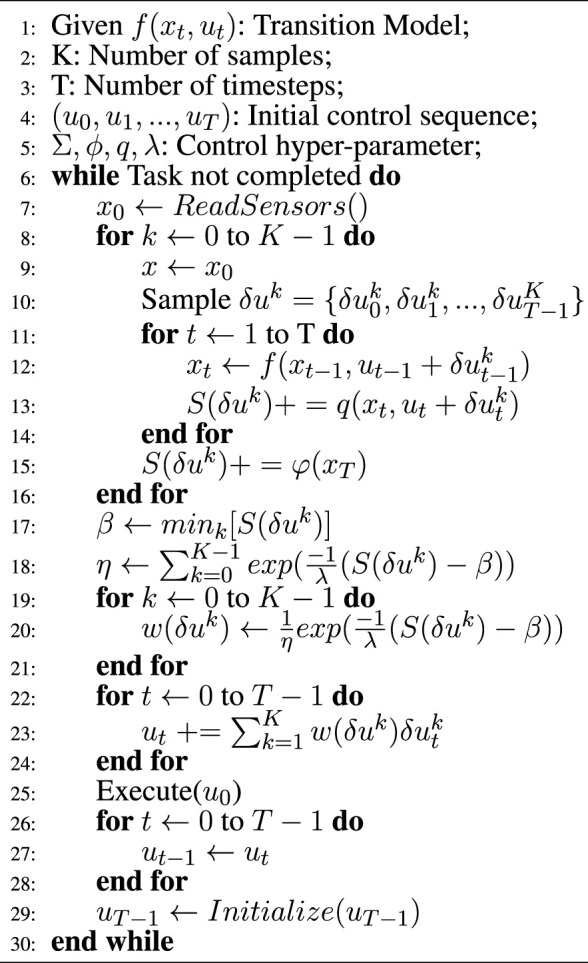

Algorithm 2 Planner-Estimator MPPI.

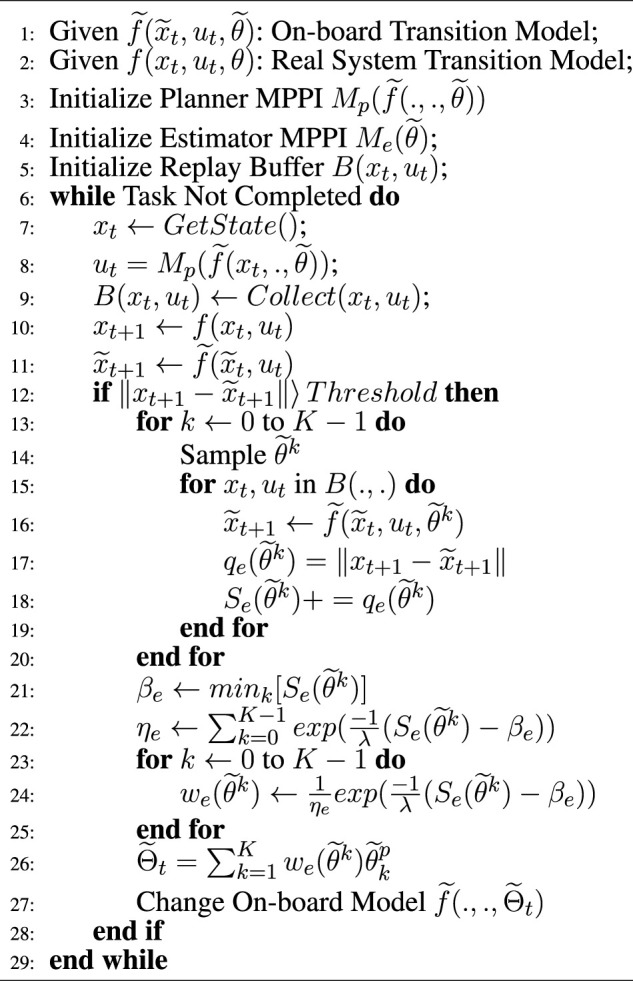

## 5 Planner-estimator MPPI

This section proposes a novel Planner-Estimator MPPI (PE-MPPI) strategy to control space robots in on-orbit debris removal missions, which can fulfill controller design requirements. First, the controller structure will be given, and lastly, the proposed algorithm will be explained.

**FIGURE 3:**
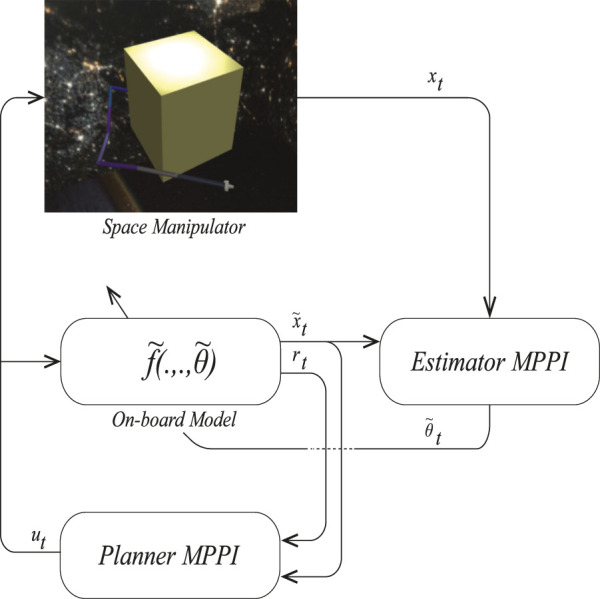
Schematic of planner-estimator MPPI

Although many studies have shown the performance of MPPI in different scenarios, its performance varies with model accuracy. To make this controller suitable for space explorations, we propose PE-MPPI to robustify the performance of MPPI against structural uncertainties. PE-MPPI is composed of two parts: Planner MPPI and Estimator MPPI. As shown in Figure 3, Planner MPPI selects the optimal control action based on the on-board model $\tilde{f}\left({\tilde{x}}_{t},u_{t},\tilde{\theta }\right)$ . The structure of Planner MPPI is the same as MPPI. It only computes the control input of the system based on the on-board model. On the other hand, Estimator MPPI attempts to estimate the model parameters and readjust the on-board model of the robot based on the norm of an error signal. In other words, whenever the on-board model fails to match the dynamic behavior of the space manipulator, Estimator MPPI estimates the model parameters and updates the model accordingly. The core idea of estimation is to sample many parameters ${\tilde{\theta }}^{k}$ from a Gaussian distribution and evaluate them as follows:

$$S_{e}\left({\tilde{\theta }}_{k}\right)=\overset{T}{\underset{t=0}{\sum }}q_{e}\left({\tilde{\theta }}_{k}\right)$$ (9)

where $q_{e}\left({\tilde{\theta }}_{k}\right)$ is the running cost for the trajectory generated with the parameter ${\tilde{\theta }}^{k}$ . Consequently, the update law of the parameters is formulated as below:

$${\tilde{\Theta }}_{t}=\frac{\sum_{k=1}^{K}\exp\left(\left(\frac{-1}{\lambda }\right)\left[S_{e,k}\left(\tau \right)-\min\left(S_{e}\right)\right]{\tilde{\theta }}_{k}\right)}{\sum_{k=1}^{K}\exp\left(\left(\frac{-1}{\lambda }\right)\left[S_{e,k}\left(\tau \right)-\min\left(S_{e}\right)\right]\right)}$$ (10)

It is important to say that the estimated model does not necessarily match the real system, but it guarantees that they would have the same response after sufficient updates.

Algorithm 2 explains PE-MPPI in detail. Based on the parameterized model $\tilde{f}\left({\tilde{x}}_{t},u_{t},\tilde{\theta }\right)$ with parameters $\tilde{\theta }$ , Planner MPPI $M_{p}\left(\tilde{f}\left(.,.,\tilde{\theta }\right)\right)$ outputs near-optimal control effort *u*_*t*_ at each time-step (code lines:7 and 8). Each response of the space robot *x*_*t*_ and the subsequent control input *u*_*t*_ is gathered in a replay buffer *B* (*x*_*t*_, *u*_*t*_) (code line: 9). The sensors of the space robot measure the response of the real system *x*_*t*+1_, while the response of the on-board model ${\tilde{x}}_{t+1}$ is calculated by the on-board model (code lines: 10 and 11). If the norm of the signal error $\left\|x_{t+1}-{\tilde{x}}_{t+1}\right\|$ is greater than a pre-defined threshold, Estimator MPPI updates the on-board model $\tilde{f}\left({\tilde{x}}_{t},u_{t},\tilde{\theta }\right)$ (line code: 12). To find the optimal parameter $\tilde{\theta }$ , many parameters ${\tilde{\theta }}^{k}$ are sampled from a Gaussian distribution, and the score of each trajectory is calculated using the running cost $q_{e}\left({\tilde{\theta }}^{k}\right)=\left\|x_{t+1}-{\tilde{x}}_{t+1}\right\|$ (code lines: 13–20). Then, the parameters of the update law are calculated, and the optimal parameters ${\tilde{\theta }}_{t}$ of the model are computed using the update law (code lines: 21–23). Finally, the on-board model is updated (code line: 27).

## 6 Simulation

**FIGURE 4:**
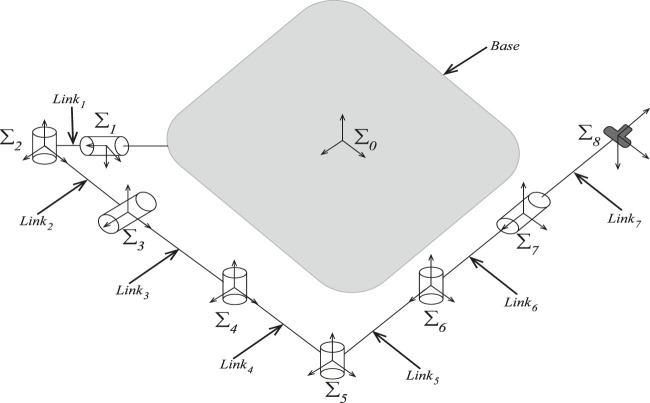
The rest configuration of the space robot and the coordinates systems of the joints.

This section investigates the performance of PE-MPPI in a MuJoCo simulation (Todorov et al., 2012) environment for a space robot (Figure 4). To analyze the performance, we consider four different scenarios, from simple to complex. The first scenario (SEN1) represents a normal operation condition with no parameter change or actuator failure. The second scenario (SEN2) represents events in which the system parameters are subject to change, while the third scenario (SEN3) represents events with actuator failure cases. The fourth scenario represents the worst operational condition in which both parameter variation and actuator failure happen concurrently. In each experiment, the space robot is planned to traverse on a *y*-axis orbit while its manipulator is commanded to approach the debris zone. To execute this mission, Planner MPPI controls six thrusters of the space base and a 7-DoF robot folded around, making a 13-dimensional control output space.

### 6.1 The general specifications of the space robot

The space robot consists of a base and a manipulator connected to the base. In non-operational conditions, the manipulator is in its resting position, folded around the base (Figure 4). However, in cases where debris is located far from the main satellite’s structure, the mission is launched to remove or catch the debris with the help of the manipulator. The 7-DoF manipulator’s length then unfolds to allow the space robot to reach far debris zones. The redundant degree of freedom assures the robot’s performance even in actuator failure conditions.

The Denavit-Hartenberg parameters of the manipulator and the inertial properties of the space robot used in this simulation are given in Tables 1, 2, respectively.

**TABLE 1:** The DH parameters of the space robot.

Joint	*α*(*rad*)	a (m)	d (m)	*θ*(*rad*)
1	$\frac{-\pi }{2}$	0.0	0.5	*θ*_1_
2	$\frac{\pi }{2}$	0.0	0.0	*θ*_2_
3	$\frac{\pi }{2}$	0.9	0.0	*θ*_3_
4	$\frac{-\pi }{2}$	0.9	0.0	*θ*_4_
5	$\frac{\pi }{2}$	0.8	0.0	*θ*_5_
6	$\frac{-\pi }{2}$	0.8	0.0	*θ*_6_
7	$\frac{\pi }{2}$	0.0	0.8	*θ*_7_

**TABLE 2:** The inertial properties of the space robot.

	Base	L1	L2	L3	L4	L5	L6	L7
*M*(*kg*)	500	20	30.0	30.0	20.0	20.0	20.0	20.0
*I*_*x*_ (*kg*.*m* ^2^)	1,400	0.1	0.25	0.25	0.25	0.25	0.25	0.25
*I*_*y*_(*kg*.*m* ^2^)	1,400	0.1	25	25	25	25	25	25
*I*_*z*_ (*kg*.*m* ^2^)	1,400	0.1	25	25	25	25	25	25

### 6.2 Operational scenarios of the space robot

#### 6.2.1 Normal operation

In normal operation, no actuator failure or system degradation occurs. Therefore, the on-board model accurately tracks the response of the real system. In this perfect situation, the spacecraft is commanded to traverse on *y*-axis while its manipulator approaches from the initial position *x*_*initial*_ = [−1.2,−1.2,0]^*T*^ to the desired target debris site *x*_*target*_ = [−2,8,0]^*T*^. The mission requirements are i) to reach the debris site, ii) to maneuver on orbit stack around axis y, and iii) to reduce control effort. Since there is no parameter uncertainties, only Planner MPPI is used. In order to meet the requirements of the mission, the cost function of Planner MPPI is designed as follows:

$$q\left(x_{t},u_{t}\right)=4\left\|x_{\mathrm{target}}-x_{\mathrm{end-effector}}\right\|+0.1\left\|u\right\|+5\left\|x_{\mathrm{base}}-x_{\mathrm{orbit}}\right\|$$ (11)

where:

*x*_*target*_: The position of the target debris site

*x*_*end−effector*_: The position of the end-effector of the manipulator

*x*_*base*_: The position of the base

*x*_*orbit*_: The position of the orbit

**FIGURE 5:**
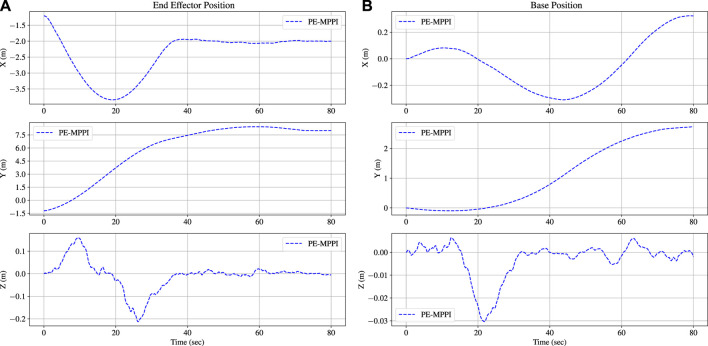
The position of the end-effector reaches the target position after 60 s in the normal operation scenario **(A)**. The space robot base position is traversed along the y-orbit **(B)**.

*u*: The control effort.The position of the end-effector relative to the inertial coordinate and the position of the space robot base are illustrated in Figure 5. After 60 s, the end-effector approaches the target site and maintains its position. The steady-state error in this mission is less than 15 cm, which is acceptable. Moreover, the space robot base position successfully tracks the orbit position, which is the *y*-axis.

#### 6.2.2 System identification

The damping coefficient of the space robot joints is assumed to differ from the on-board model parameters in the second scenario. The difference between the model and the real system can result in poor approaching behavior. Hence, adopting a strategy to identify the system’s parameters in real-time is crucial in this mission. Thus, both Planner MPPI and Estimator MPPI are used. Since the goal of the mission is the same as the normal operation scenario, the cost function of the Planner MPPI is the same. On the other hand, the running cost function of Estimator MPPI is defined as below:

$$q\left({\tilde{\theta }}_{k}\right)=2\left\|x_{t+1}-{\tilde{x}}_{t+1}\right\|$$ (12)

**FIGURE 6:**
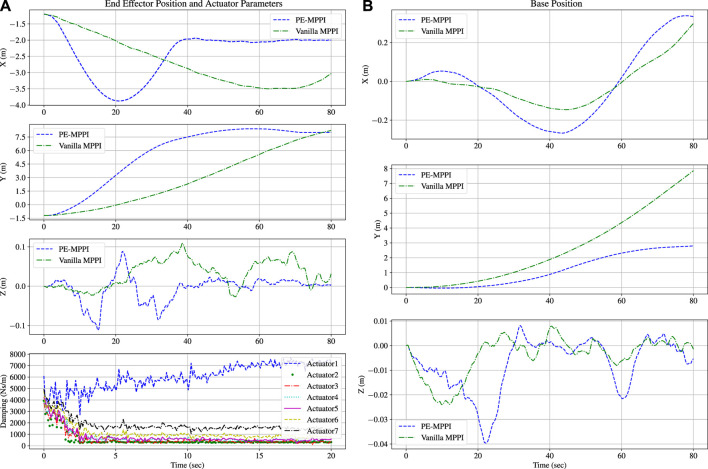
System identification scenario; Comparison between PE-MPPI and vanilla MPPI for the end-effector position **(A; Top)**. Convergence of the parameters of the on-board model to real system **(A; Bottom)**. Comparison between PE-MPPI and vanilla MPPI for the space robot base position **(B)**.

The damping coefficients of the on-board model were set to $5000\frac{Ns}{m}$ at the beginning of the simulation, while the damping coefficients of the real system were one-tenth of the damping coefficients of the on-board model. A comparison between the performance of PE-MPPI and vanilla MPPI applied to the model with parameter uncertainties is illustrated in Figure 6A. Moreover, the convergence of damping coefficients is depicted in Figure 6A. PE-MPPI can reach the target position in the system identification mission after 70 s. In contrast, the performance of vanilla MPPI deteriorates due to the lack of a mechanism for adjusting the parameters of the model. All parameters converge to the real system parameters after 20 s, while there is a significant error in estimating the first and last parameters. However, these errors have little impact on system performance as the end-effector can reach the debris site after 70 s. It can be concluded that estimating the parameters $\tilde{\theta }$ increases the stability of the system and reduces the steady-state error resulting in better performance. In addition, as is shown in Figure 6B, for both PE-MPPI and vanilla MPPI, the space robot base position is traversed on the *y*-axis. Since the parameter uncertainties are related to the joint parameters, the parameter uncertainties mainly affect the end-effector position rather than the base position.

#### 6.2.3 Actuator failure

Due to many sources of failure in the space missions, such as debris collision or system degeneration, actuator failure can happen during the robot’s lifespan. The main challenge is that the system dynamics will change suddenly, resulting in instability and poor performance. In this critical condition, the source of failure is well understood; hence parameter estimation is not required and Estimator MPPI is not used. However, adopting a robust and adaptable control strategy, which can alter in real-time, is required to guarantee the system’s stability with minimum human intervention. The cost of Planner MPPI is the same as the two previous scenarios.

**FIGURE 7:**
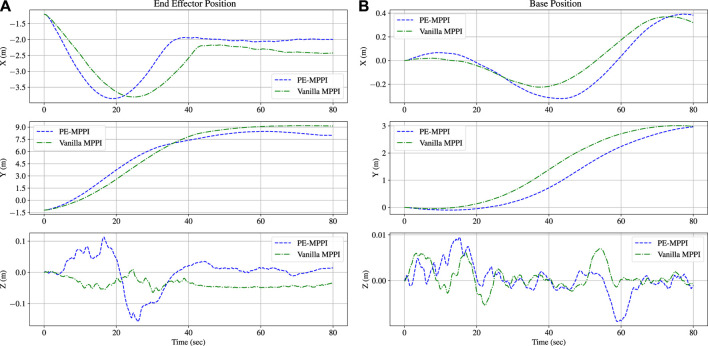
Comparison between PE-MPPI and vanilla MPPI in the actuator failure scenario for the end-effector position **(A)** and the space robot base position **(B)**.

In the third scenario, the space robot will lose one of its degrees of freedom, and consequently, this actuator cannot be controlled anymore (the second actuator is chosen to be locked). The performance of PE-MPPI is compared to vanilla MPPI in which the on-board model is not changed by actuator failure. As shown in Figure 7, a lack of updating mechanism for the on-board model in vanilla MPPI causes poor performance compared to PE-MPPI, and it can conveniently update its model and successfully reach the target position and remain at this position after 60 s. Moreover, the base position is traversed on the *y*-axis. Similar to the system identification scenario, since actuator failure is mainly related to the joint space, it affects the end-effector position more than the base position.

#### 6.2.4 System identification and actuator failure

In the last and worst scenario, both actuator failure and system parameter change occur simultaneously. In this condition, the estimator section would help the planner to control the space robot and reach the desired position while the failed actuator (the third actuator is chosen) is locked. The cost function of PE-MPPI is the same as the system identification scenario. Similar to the second scenario, all damping coefficients were initialized to be $5000\frac{Ns}{m}$ while the real system parameters were one-tenth of the on-board model.

**FIGURE 8:**
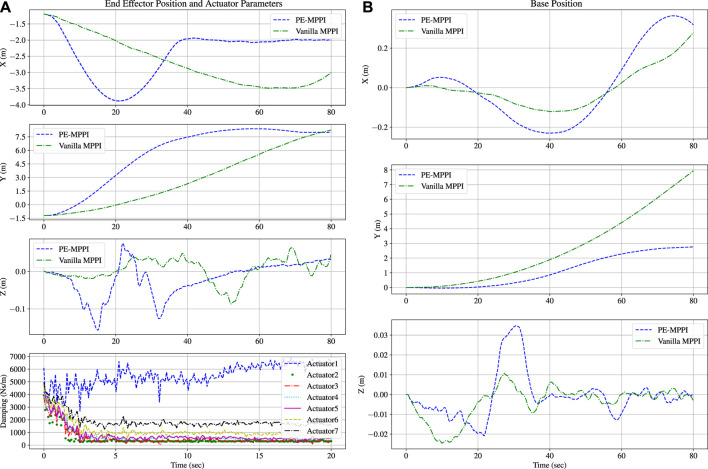
System identification and actuator failure scenario; Comparison between PE-MPPI and vanilla MPPI for the end-effector position **(A; Top)**. Convergence of the parameters of the on-board model to the real system **(A; Bottom)** Comparison between PE-MPPI and vanilla MPPI for the space robot base position **(B)**.

**FIGURE 9:**
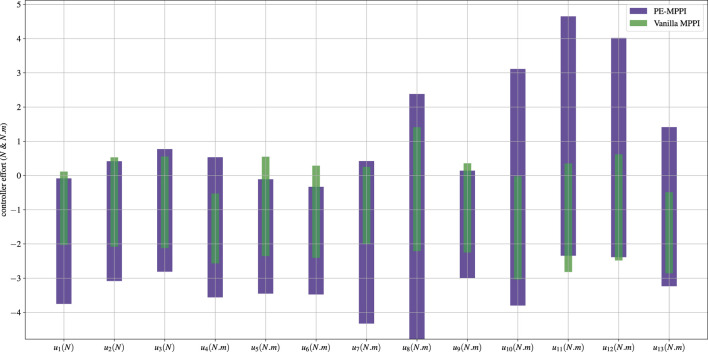
The space robot control effort bounds in the system identification *and* actuator failure scenario.

As shown in Figure 8A, after 20 s, all parameters converged to the real system parameters, while there was a significant error in estimating the first and last parameters. The estimated parameters $\tilde{\theta }$ showed more fluctuation compared to the system identification scenario, indicating the combination of events could reduce the controller’s performance in both estimating parameters and steady-state error. Moreover, PE-MMPI takes more time to reach the target position (after 70 s), while vanilla MPPI cannot accomplish the mission (Figure 8A). In addition, the base position successfully traverses on the *y*-axis (Figure 8B). Figure 9 shows the bounds of the control effort of both PE-MPPI and vanilla MPPI for the system identification and actuator failure scenario. As it is expected, PE-MPPI needs more control effort than vanilla MPPI, since it manages parameter uncertainties and actuator failure.

## 7 Conclusion

This study proposed a novel Planner-Estimator MPPI (PE-MPPI) algorithm to control space robots in debris removal pre-capture phase missions subject to system malfunctioning and structured parameter changes. Four scenarios were considered for testing the controller’s performance: normal operation, system identification, actuator failure, and combined system identification and actuator failure. In each scenario, the performance of PE-MPPI is compared to vanilla MPPI. Results proved the superiority of the proposed algorithm over vanilla MPPI, especially in the fourth scenario, where the combination of events results in poor performance. It was shown that PE-MPPI could maintain its performance in different scenarios, with negligible degeneration compared to normal operation. Furthermore, the estimator assures that the on-board model tracks the real system, while some errors are in estimating parameters (especially the first and last actuators’ damping coefficient). It is worth mentioning that the convergence of damping coefficients to their real values is not guaranteed, but the norm of difference signal would be minimized.

## Data Availability

The original contributions presented in the study are included in the article/supplementary material, further inquiries can be directed to the corresponding author.
